# Electroacupuncture Improves Trinitrobenzene Sulfonic Acid-Induced Colitis, Evaluated by Transcriptomic Study

**DOI:** 10.1155/2014/942196

**Published:** 2014-06-09

**Authors:** Tin-Yun Ho, Hsin-Yi Lo, De-Cheng Chao, Chia-Cheng Li, Jau-Jin Liu, Chingju Lin, Chien-Yun Hsiang

**Affiliations:** ^1^Graduate Institute of Chinese Medicine, China Medical University, 91 Hsueh-Shih Road, Taichung 40402, Taiwan; ^2^Graduate Institute of Cancer Biology, China Medical University, 91 Hsueh-Shih Road, Taichung 40402, Taiwan; ^3^Department of Microbiology, China Medical University, 91 Hsueh-Shih Road, Taichung 40402, Taiwan; ^4^Department of Physiology, China Medical University, 91 Hsueh-Shih Road, Taichung 40402, Taiwan

## Abstract

Inflammatory bowel disease is a chronic colonic inflammation that displays symptoms like diarrhea and weight loss. Acupuncture has been widely accepted by Western countries for the treatment of pain. Here, we analyzed efficacy and mechanism of electroacupuncture (EA) on trinitrobenzene sulfonic acid- (TNBS-) induced colitis in mice. Mice were intrarectally administered with 250 mg/kg TNBS and electroacupunctured at Quze (PC3) and Neiguan (PC6) acupoints, which have been applied for gastrointestinal disorders. Gene expression profiles in colons and spleens were analyzed by microarray for the elucidation of mechanism of EA. Our data showed that EA at PC3 and PC6 improved macroscopic and microscopic features of colitis and the improvement displayed a frequency-dependent manner. Administration of TNBS upregulated the expression of most cytokine genes in colons, while EA downregulated the expression of TNBS-induced cytokine genes. Pathway analysis showed that EA significantly affected inflammatory pathways in colons and immunity-associated pathway in spleens. Immunohistochemical staining further showed that EA decreased the expression of interleukin-1**β** and nuclear factor-**κ**B. In conclusion, this is the first study reporting the global gene expression profiles of EA on TNBS-induced colitis. Our findings suggested that inflammatory and immunity pathways were involved in the anti-inflammatory mechanism of EA on colitis induced by TNBS.

## 1. Introduction

The incidence and prevalence of inflammatory bowel diseases (IBD), including Crohn's disease (CD) and ulcerative colitis (UC), are about 0.5 to 1.0% of the population in the United States and the United Kingdom. An elevating incidence in the Asia-Pacific region is also found [1, 2]. IBD is an idiopathic chronic inflammation that presents symptoms like diarrhea and suddenly weight loss. The etiology of IBD remains unclear, but the imbalance of mucosal homeostasis plays an important role in the pathogenesis of IBD [3]. Anti-inflammatory drugs, such as 5-aminosalicylic acid (5-ASA) and immunosuppressants, such as antitumor necrosis factor (TNF) agents, have been used for the treatment of IBD [2, 4]. However, a long-term treatment of colitis by 5-ASA and anti-TNF displays intolerance and side effects, such as nausea, allergy, pancreatitis, and hepatitis [5, 6]. IBD patients may turn to complementary and alternative medicine because they are unresponsive or suffer from sides effects during conventional treatment [7].

Acupuncture, one of the oldest healing practices, has been widely accepted by the US Food and Drug Administration and the World Health Organization [8]. Clinical evidences have shown the efficacy of acupuncture on the postoperative analgesia and anesthesia [9]. Moreover, acupuncture is effective for the treatment of certain symptoms and diseases, such as chronic pain syndrome, osteoarthritis, and neurological diseases [10–14]. Clinical studies have also shown that acupuncture improves pain and functional activity in patients [15].

In this study, we evaluated the efficacy and mechanism of electroacupuncture (EA) on trinitrobenzene sulfonic acid- (TNBS-) induced colitis in mice. Acupoints Quze (PC3) and Neiguan (PC6) were selected because these acupoints have been applied for the treatment of gastrointestinal disorders [16]. Gene expression profiles of colons and spleens were analyzed by microarray to provide a whole picture view of the biological pathways on the improvement of TNBS-induced colitis by EA.

## 2. Materials and Methods

### 2.1. Materials

TNBS was purchased from Sigma (St. Louis, MO, USA) and dissolved in ethanol. Mouse monoclonal antibody against nuclear factor-*κ*B (NF-*κ*B) and rabbit polyclonal antibody against interleukin-1*β* (IL-1*β*) were purchased from Chemicon (Temecula, CA, USA) and Santa Cruz (Santa Cruz, CA, USA), respectively.

### 2.2. Induction of Colitis

Inbred female BALB/c mice (6–8 weeks old, 21 ± 2 g weight) were purchased from National Laboratory Animal Center (Taipei, Taiwan). Mouse experiments were conducted under ethics approval from China Medical University Animal Care and Use Committee (Permit Number: 101-61-N).

Induction of colitis was performed as described previously [17, 18]. Briefly, mice were slowly administered with TNBS (250 mg/kg in 50% ethanol, total volume 0.1 mL) into the colons of lightly anesthetized mice via a thin catheter attached to a 0.5 mL syringe. Mice that were given 0.1 mL of 50% ethanol solution were used as mock.

### 2.3. Treatment of EA

**Figure 1:**
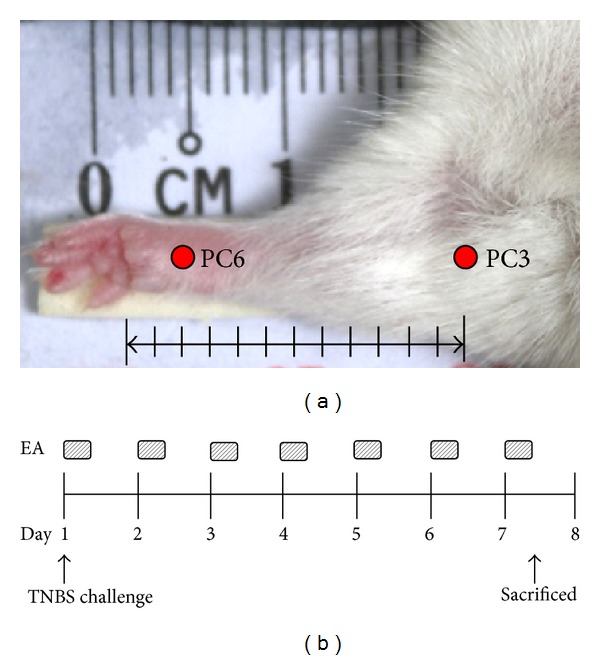
Experimental protocol. (a) Location of PC3 and PC6 acupoints. (b) Experimental design. Mice were given TNBS once and then electroacupunctured at PC3 and PC6 acupoints for 20 min once a day. EA was performed for consecutive 7 days. Mice were sacrificed 7 days after TNBS administration.

EA was performed at two acupoints PC3 and PC6 (Figure 1(a)). PC3 is located at the midpoint of cubital crease. It is the ulnar side of the tendon of biceps muscle. PC6 is located at the palmar side of forearm, which is approximately 3 mm above the crease of wrist. Mice were anesthetized with isoflurane. Stainless steel needles (diameter 0.235 mm, length 1.27 cm) were inserted into PC3 and PC6 acupoints, at a depth of 1 mm. After insertion, needle handles were connected to EA apparatus (MH-350 low-frequency electric therapy machine, Ming Horng Industrial Co., Kaohsiung, Taiwan) and a constant electrical stimulus (frequency 1, 10, or 100 Hz) was applied for 20 min once a day for consecutive 7 days (Figure 1(b)).

A total of forty mice were randomly divided into five groups with eight mice. In mock group, mice were given 0.1 mL of 50% ethanol. In TNBS group, mice were given 0.1 mL of 250 mg/kg TNBS in 50% ethanol. In EA-1, EA-10, and EA-100 groups, mice were given 0.1 mL of 250 mg/kg TNBS in 50% ethanol and electroacupunctured at PC3 and PC6 with a pulse of 1 Hz, 10 Hz, and 100 Hz frequency, respectively. Mice were sacrificed 7 days after TNBS administration for colitis assessment, RNA extraction, and immunohistochemical evaluations.

### 2.4. Assessment of Colitis

The severity of colitis was assessed by colonic weight/length ratio and macroscopic and histological features. Macroscopic assessment was graded as follows: 0, no ulcer and no inflammation; 1, local hyperaemia without ulceration; 2, ulceration without hyperaemia; 3, ulceration and inflammation at one site only; 4, two or more sites of ulceration and inflammation; 5, ulceration extending more than 2 cm. For microscopic analysis, colons were fixed, sectioned, and stained with hematoxylin/eosin. Histological changes were graded as follows: 0, no sign of inflammation; 1, very low level of leukocyte infiltration; 2, low level of leukocyte infiltration; 3, high level of leukocyte infiltration, high vascular density, and thickening of the colon wall; 4, transmural infiltration, loss of goblet cells, high vascular density, and thickening of the colon wall. Macroscopic and microscopic scores were assessed by three experts in a blinded fashion.

### 2.5. Microarray Analysis

Total RNAs were extracted from colons and spleens using RNeasy Mini kit (Qiagen, Valencia, CA, USA). Microarray analysis was performed as described previously [16, 19]. Briefly, Cy5 fluorescence-labeled RNA targets were hybridized to the Mouse Whole Genome OneArray (Phalanx Biotech Group, Hsinchu, Taiwan). Number of replicates was two. The fluorescent intensity of each spot was analyzed by GenePix 4.1 software (Molecular Devices, Sunnyvale, CA, USA). The signal intensity of each spot was corrected by subtracting background signals in the surrounding. We filtered out spots in which signal-to-noise ratio was less than 1 or control probes. Spots that passed these criteria were normalized by the R program in limma package. The fold changes of genes in TNBS group were calculated by dividing the normalized signal intensities of genes in TNBS-treated mice by those in mock. The fold changes of genes in EA-100 group were calculated by dividing the normalized signal intensities of genes in 100 Hz-treated mice by those in TNBS-treated mice. Genes with fold changes ≥2 or ≤−2 in colon and spleen were grouped into Kyoto Encyclopedia of Genes and Genomes (KEGG) pathways (http://www.genome.jp/kegg/pathway.html). We used the geneSetTest function implemented in the R program to test the significant KEGG pathways. Microarray data are MIAME compliant and raw data have been deposited in the Gene Expression Omnibus (Accession Number: GSE57771).

### 2.6. Immunohistochemical Staining (IHC)

Paraffin-embedded colonic sections were deparaffinized in xylene and rehydrated in graded alcohol. After quenching the endogenous peroxidase and blocking the nonspecific binding, sections were incubated with mouse monoclonal antibody against NF-*κ*B p65 subunit at 1 : 100 dilution or rabbit polyclonal antibody against IL-1*β* at 1 : 50 dilution overnight at 4°C. Sections were then incubated with biotinylated secondary antibody at room temperature for 20 min, incubated with avidin-biotin complex reagent, and stained with 3,3′-diaminobenzidine (Histostain-Plus, Invitrogen, Camarillo, CA, USA).

### 2.7. Statistics Analysis

Data were presented as mean ± standard error. Student's *t*-test was used for a comparison between two experiments. A value of *P* < 0.05 was considered statistically significant.

## 3. Results

### 3.1. EA at PC3 and PC6 Acupoints Improved TNBS-Induced Colitis in Mice

**Figure 2:**
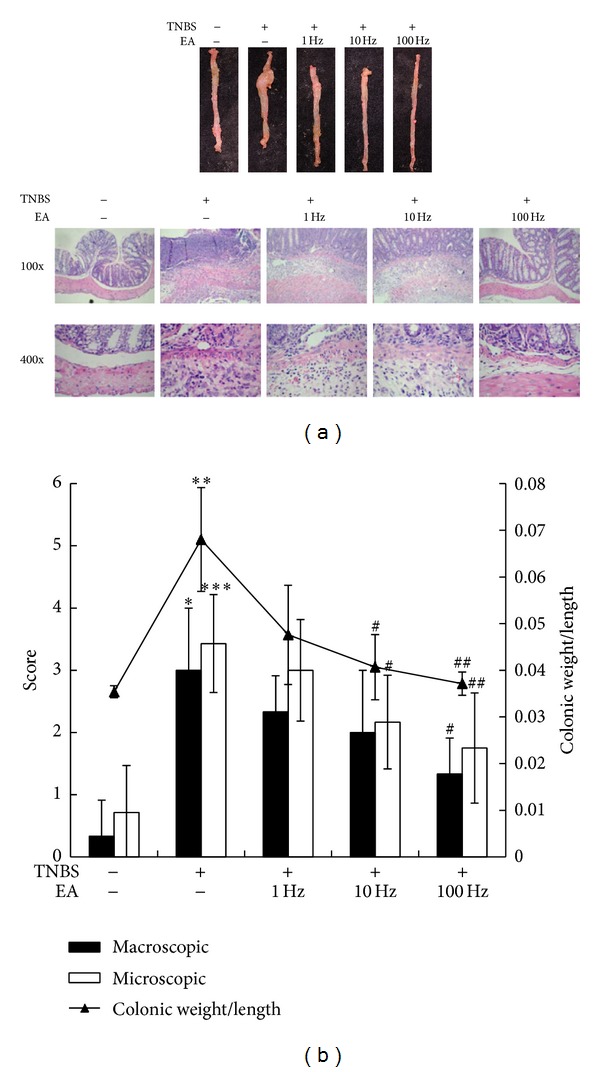
Effect of EA on TNBS-induced colitis in mice. Mice were intrarectally administered with 250 mg/kg TNBS once and/or various frequencies of EA for 20 min daily. Seven days later, mice were sacrificed and examined for colitis by macroscopic lesion and microscopic sections. (a) Macroscopic and microscopic features of colons. Photos are representative images (*n* = 8/group). (b) Quantification of colitis severity. Values are mean ± standard error (*n* = 8/group). *P < 0.05, **P < 0.01, and ***P < 0.001, compared with mock. ^#^ *P* < 0.05, ^##^ *P* < 0.01, compared with TNBS.

Previous studies have demonstrated that intrarectal administration of TNBS in mice induces colitis resembling IBD in human [20]. Our previous studies have also shown that intrarectal administration of 250 mg/kg TNBS significantly induces macroscopic and microscopic damages in colons [17, 18]. Therefore, the efficacy of EA on TNBS-induced colitis was evaluated by the administration of 250 mg/kg TNBS into colons and EA at PC3 and PC6 acupoints. As shown in Figure 2, no sign or a very low level of macroscopic and microscopic lesions in the colons was observed in mock group. However, macroscopic and microscopic examinations of colons after TNBS induction showed inflammation and leukocyte infiltration. The colonic weight/length ratio of TNBS induction was also significantly increased. In contrast, EA at PC3 and PC6 acupoints significantly improved the macroscopic and microscopic features and colonic weight/length ratio of TNBS-induced colitis. And the improvement of EA displayed a frequency-dependent manner. These findings suggested that EA at PC3 and PC6 acupoints ameliorated the colitis induced by TNBS in mice.

### 3.2. Analysis of EA-Affected Gene Expression Profile in Colonic Tissues

Increased expression of cytokines, such as IL-1*β*, IL-6, TNF-*α*, and interferon-*γ* (IFN-*γ*), is observed after TNBS treatment, suggesting that cytokines are involved in the pathogenesis of TNBS-induced colitis [18]. To elucidate the mechanism of EA on the improvement of TNBS-induced colitis, we performed microarray analysis and evaluated the expression of cytokine genes in TNBS and/or EA treatment. In a total of 29,922 genes, 839 and 735 transcripts were upregulated with fold change ≥2 and downregulated with fold change ≤−2, respectively, by TNBS. In contrast, 1,519 and 1,334 transcripts were upregulated and downregulated, respectively, by EA treatment. The expression levels of cytokine genes in colonic tissues treated with TNBS and/or EA are shown in Table 1. It was interesting to find that administration of TNBS upregulated the expression of most cytokine genes, while EA downregulated the expression of TNBS-induced cytokine genes. These findings suggested the crucial roles of cytokines in the pathogenesis of TNBS-induced colitis and the amelioration of EA on TNBS-induced colitis.

### 3.3. Analysis of EA-Affected Gene Expression Profile in Spleens

In addition to the colonic tissues, we wondered whether TNBS and EA treatment affected the immune responses of distant immune systems. The gene expression profile of spleens was therefore analyzed by microarray. In a total of 29,922 genes, 86 and 83 transcripts were upregulated by TNBS and EA, respectively, while 39 and 112 transcripts were downregulated by TNBS and EA, respectively. Genes with fold changes ≥2 or ≤−2 were further selected for pathway analysis. As shown in Table 2, inflammation-associated pathways, such as IL-1, IL-11, TNF, and tumor growth factor-*β* (TGF-*β*) signaling pathways, were significantly affected in colonic tissues by EA, while immunity-associated pathways, such as B cell receptor, Toll-like receptor, and T cell receptor signaling pathways, were significantly regulated in spleens by EA treatment. The symbols and fold changes of genes in each pathway have been shown on Supplementary Table 1 (see Supplementary Table 1 in Supplementary Materials available online at http://dx.doi.org/10.1155/2014/942196). These findings suggested that EA might regulate the inflammatory pathways in colons and immunity pathways in spleens, and, in turn, improve the colitis induced by TNBS.

### 3.4. The Involvement of NF-*κ*B and IL-1*β* in the Improvement of EA on TNBS-Induced Colitis

**Figure 3:**
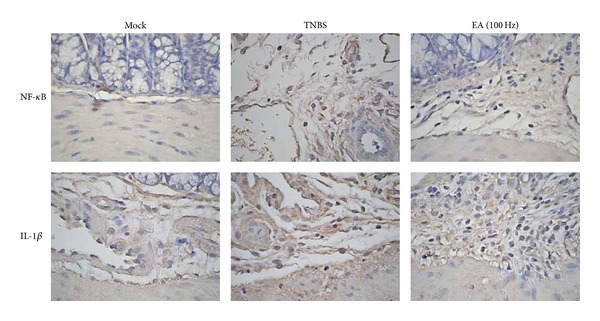
Effect of EA on the NF-*κ*B and IL-1*β* expression in colons by IHC. Mice were administered with TNBS once and/or 100 Hz EA for 20 min daily. Seven days later, mice were sacrificed and colons were stained with antibodies against NF-*κ*B and IL-1*β* (400x magnification). Photos are representative images (*n* = 8/group).

Our findings indicated that IL-1 and IL-1*β* signaling pathways were significantly affected by EA in colonic tissues. Moreover, NF-*κ*B is a well-known transcription factors that regulates the expression of cytokines [21]. Therefore, we performed IHC to evaluate the expression of IL-1*β* and NF-*κ*B in colonic tissues treated with EA. As shown in Figure 3, there were many brown NF-*κ*B or IL-1*β*-reactive cells in TNBS-treated colons, while the areas of NF-*κ*B or IL-1*β*-reactive regions in colons were decreased by EA. These findings suggested the involvement of IL-1*β* and NF-*κ*B in the amelioration of TNBS-induced colitis by EA.

## 4. Discussion

Acupuncture that penetrates the skin with needles and stimulates certain points on the body is one of the oldest healing practices in the world. EA is an advanced modification of traditional acupuncture that is accepted by Western countries because of its reproducibility [8]. According to the principles of traditional Chinese medicine, stimulation of specific acupuncture points remedies imbalances of energy flow within these meridians [22]. Moreover, after the practice for centuries, acupuncture has shown the efficacies on the treatment of nausea/vomiting, the reduction of stress, the relief of pain, and the enhancement of immunity [10–14, 23]. There is an increasing use of EA in patients with IBD worldwide. Approximately 7% of patients with IBD in France and 9.8% of IBD patients in Taiwan apply EA for the treatment of IBD [7, 24]. Efficacies of acupuncture on the treatment of IBD have been evaluated previously [25]. A randomized controlled study with 51 patients with mild to moderate active CD showed that acupuncture offers an additional therapeutic benefit in patient [24]. Another randomized controlled study with 29 patients with mild to moderate active UC showed that a significant decrease in mean colitis active index after treatment is observed [25]. However, the mechanism of EA on IBD remains to be clarified.

There are approximately 700 acupoints on the body. Some acupoints have displayed anti-inflammatory potentials. For examples, acupuncture at Zusanli (ST36) inhibits the expression of IL-1*β*, IL-6, and TNF-*α* mRNA and ameliorates the experimental UC in rats [26, 27]. EA at Shangjuxu (ST37) restores cytokine homeostasis by the decreased IL-1*β* and the increased IL-4, and, in turn, improves UC in rats [28]. EA at Qihai (RN6) and Tianshu (ST25) improves UC in rats via the regulation of immunological function, the reduction of inflammatory cytokine expressions, and the promotion of neutrophil apoptosis [29]. Moreover, EA at Hoku (LT4) and ST36 displays a beneficial effect on colitis in rats via *β*-adrenoceptor activation [30].

In this study, we analyzed the effects of PC3 and PC6 on TNBS-induced colitis in mice. Acupuncture at PC3 and PC6 has been applied for chronic angina, arrhythmia, chest pain, and gastrointestinal disorders, such as vomiting, diarrhea, and nausea in traditional Chinese medicine. EA at PC6 decreases the levels of plasma nitrogen oxide and TNF-*α* in endotoxin shock rats [31]. Pretreatment of EA at PC6 decreases plasma TNF-*α* and IL-1*β* and decreases plasma nitrite, renal inducible nitric oxide synthase, and NF-*κ*B, resulting in the attenuation of lipopolysaccharide-induced inflammatory response and mitigated acute renal injury [32]. In this study, we found that EA at PC3 and PC6 downregulated the expression of TNBS-induced cytokine genes, such as IL-1*β*, IL-6, IL-13, and IL-18 genes, and significantly affected IL-1 and TGF-*β* signaling pathways in colons. Overproduction of proinflammatory cytokines, including TNF-*α*, IL-1*β*, and IL-6, is observed in several animal colitis models [17, 18, 33]. High levels of proinflammatory cytokines in the mucosa might lead to the excessive production of matrix degrading enzymes by gut fibroblasts and the loss of mucosa integrity and ulceration [34]. Additionally, IL-13 is produced by CD4+ T cells and natural killer cells, and IL-18 is specifically required for the* in vivo* regulation of IL-13 production. IL-13 acts on the IL-13 receptor and induces TGF-*β*1 production, resulting in fibrosis, one of the major macroscopic features of IBD [33]. Therefore, our data suggested that EA at PC3 and PC6 might inhibit the production of cytokines and improve the mucosa integrity, ulceration, and thickening of the colonic wall, resulting in the amelioration of TNBS-induced colitis.

**Figure 4:**
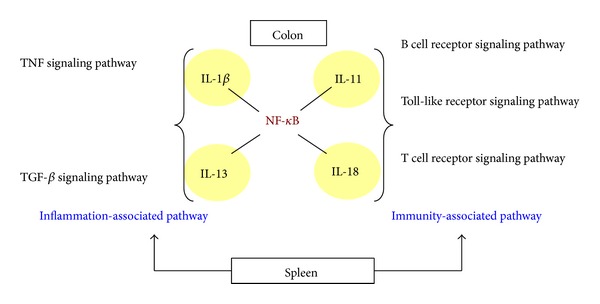
Schematic diagram illustrates the anti-inflammatory mechanism of EA at PC3 and PC6 in TNBS-induced colitis.

According to the collateral meridian therapy [35, 36], needling at PC3 and PC6 on the pericardium meridian allows to divert the obstructed Qi from stomach meridian to pericardium meridian, leading to the amelioration of inflammation and the balance between meridians and immune systems. Therefore, in addition to colonic tissues, we also evaluated the gene expression profiles of spleen in TNBS-induced colitis. KEGG pathway analysis showed that immunity-associated pathways, such as B cell receptor, Toll-like receptor, and T cell receptor signaling pathways, in spleen were significantly regulated by EA treatment, while inflammatory pathways in colons were affected by EA. Because NF-*κ*B is a critical transcription factor that regulates the expression of cytokine genes and our data showed that EA suppressed the TNBS-induced NF-*κ*B in the colon, we speculated that EA at PC3 and PC6 inhibited the expression of TNBS-induced cytokines via the suppression of NF-*κ*B and affected immunity and inflammatory pathways, leading to the improvement of TNBS-induced colitis (Figure 4).

## 5. Conclusion

In conclusion, this is the first report describing the global gene expression profiles of EA on TNBS-induced colitis. Our findings showed that EA at PC3 and PC6 ameliorated colitis induced by TNBS. Moreover, the improvement might result from the regulation of cytokine expressions and inflammatory and immunity-associated pathways in colons and spleens.

## Supplementary Material

Supplementary Table 1 shows that EA affected the expression of genes involved in inflammation-associated pathways in colons and affected the expression of genes involved in immunity pathways in spleens.

## Figures and Tables

**Table 1:** Expression levels of cytokine genes in colonic tissues treated with TNBS and/or EA (100 Hz) at PC3 and PC6 acupoints.

Gene	Fold changes	
	TNBS^a^	EA^b^
Interleukin-1*α*	1.18 ± 0.64	−1.50 ± 0.30
Interleukin-1*β*	1.34 ± 0.54	−1.84 ± 0.10
Interleukin-3	1.07 ± 0.09	−1.04 ± 0.05
Interleukin-4	1.02 ± 0.04	1.01 ± 0.05
Interleukin-6	1.54 ± 0.88	−1.66 ± 0.34
Interleukin-7	1.10 ± 0.06	−1.17 ± 0.04
Interleukin-9	1.02 ± 0.13	−1.16 ± 0.07
Interleukin-10	−1.15 ± 0.17	1.00 ± 0.09
Interleukin-11	2.87 ± 2.64	−1.84 ± 0.54
Interleukin-13	5.72 ± 6.34	−3.59 ± 0.30
Interleukin-15	−2.28 ± 0.23	−1.16 ± 0.40
Interleukin-16	1.04 ± 0.01	−1.09 ± 0.04
Interleukin-17B	1.03 ± 0.05	−1.12 ± 0.06
Interleukin-18	3.11 ± 3.44	−4.34 ± 0.23
Interleukin-20	1.05 ± 0.02	−1.08 ± 0.01
Interleukin-24	1.02 ± 0.05	−1.06 ± 0.06
Interleukin-25	1.14 ± 0.54	−1.49 ± 0.25
Interferon-*β*	1.07 ± 0.14	−1.03 ± 0.09
Interferon-*γ*	1.20 ± 0.13	−1.70 ± 0.09
Tumor necrosis factor-*α*	1.08 ± 0.07	−1.09 ± 0.07

^a^Fold changes of cytokine genes were calculated by dividing the normalized signal intensities of genes in TNBS-treated colitis by those in mock.

^b^Fold changes of cytokine genes were calculated by dividing the normalized signal intensities of genes in EA-treated TNBS-induced colitis by those in untreated TNBS-induced colitis.

**Table 2:** Pathway analysis of genes in colonic tissues and spleens treated with TNBS and EA (100 Hz) at PC3 and PC6 acupoints.

Pathways^a^	*P* value^b^	
	Colonic tissue	Spleen
Inflammation-associated pathways		
IL-1 signaling pathway	0.00251	0.00841
IL-1*β* signaling pathway	0.00567	0.08017
Interleukin signaling pathway	0.01007	0.06366
IL-11 signaling pathway	0.01198	0.28622
TGF-*β* signaling pathway	0.00118	0.00014
TNF signaling pathway	0.06086	0.00077
Immunity-associated pathways		
B cell receptor signaling pathway	0.13946	2.88 × 10^−9^
Toll-like receptor signaling pathway	0.00295	9.69 × 10^−7^
T cell receptor signaling pathway	0.34349	5.73 × 10^−6^

^a^Pathways which were associated with inflammation and immunity and were affected significantly either in colons or spleens are shown.

^b^ *P* values were calculated by the geneSetTest function implemented in the limma package.
